# Micro-Size Layers Evaluation of CIGSe Solar Cells on Flexible Substrates by Two-Segment Process Improved for Overall Efficiencies

**DOI:** 10.3390/molecules30030562

**Published:** 2025-01-26

**Authors:** Jiajer Ho, Da-Ming Yu, Jen-Chuan Chang, Jyh-Jier Ho

**Affiliations:** 1 Institute of Interdisciplinary Computing & the Arts, University of California San Diego, 9500 Gilman Drive, La Jolla, San Diego, CA 92093, USA; jjh10422@gmail.com; 2 Electrical Engineering Department, National Taiwan Ocean University, Keelung 20224, Taiwan; ffrraannkk33@gmail.com; 3 Green Energy & Environment Research Laboratories, Industrial Technology Research Institute, Hsinchu 310401, Taiwan; charn122@gmail.com

**Keywords:** Cu-(In, Ga)-Se_2_ (CIGSe) solar cells, two-segment process, metal alloy composition, internal and external quantum efficiency (IQE/EQE), sustainable goal, eco-friendly community

## Abstract

This paper details the enhancement of the optoelectronic properties of Cu-(In, Ga)-Se_2_ (CIGSe) solar cells through a two-segment process in the ultraviolet (UV)–visible spectral range. These include fine-tuning the DC sputtering power of the absorber layer (ranging from 20 to 40 W at segment I) and thoroughly checking the trace micro-chemistry composition of the absorber layer (CdS, ZnO/CdS, ZnMgO/CdS, and ZnMgO at segment II). After segment I of treatment, the optimal 30 W CIGSe absorber layer (i.e., with a 0.95 CGI ratio) can be obtained, it can be seen that the Cu-rich film exhibits the ability to significantly promote grain growth and can effectively reduce its trap state density. After the segment II process aimed at replacing toxic CdS, the optimal metal alloy (Zn_0.9_Mg_0.1_O) composition (buffer layer) achieved the highest conversion efficiency (*η*) of 8.70%, also emphasizing its role in environmental protection. Especially within the tunable bandgap range (2.48–3.62 eV), the developed overall internal and external quantum efficiency (IQE/EQE) is significantly improved by 13.15% at shorter wavelengths. A photovoltaic (PV) module designed with nine optimal CIGSe cells demonstrated commendable stability. Variation remained within ±5% throughout the 60-day experiment. The PV modules in this study represent a breakthrough benchmark toward a significant advance in the scientific understanding of renewable energy. Furthermore, this research clearly promotes the practical application of PV modules, harmonizes with sustainable goals, and actively contributes to the creation of eco-friendly communities.

## 1. Introduction

Within photovoltaic (PV) applications, copper indium gallium selenide (Cu(In,Ga)Se_2_, CIGSe) emerges as a standout absorber material due to its nearly optimal bandgap, high optical absorption coefficients, and long-term stability [1]. Numerous studies have documented heightened photo-sensor efficiency achieved by coupling a CIGSe absorber with an array of diverse buffer layer materials [2,3,4,5]. Within a CIGSe structure, as light penetrates the absorber layer, photons carrying energies corresponding to the bandgap values can be efficiently absorbed by the layer, minimizing light loss [6]. The bandgap of CuIn_1−*x*_Ga*_x_*Se demonstrates its minimum value (*E*g = 1.02 eV) at *x* = 0 and its maximum value (*E*g = 1.68 eV) at *x* = 1 [1]. The inherent defects significantly influence both the structure and physical characteristics of CIGSe films [7], emphasizing the crucial role of minimizing stoichiometry deviation during the film fabrication process. To achieve a high-quality single-phase chalcopyrite film, the optimization of process parameters, such as sputtering powers, is essential. However, there is an increasing need to prioritize environmental and human health concerns. Despite a high efficiency of 23.82% achieved by the buffer-CdS material in CIGSe photo-sensors [3], these Cd-based materials pose environmental hazards, triggering valid concerns regarding ecological impact.

Nowadays, the impact on environmental issues and human health has recently been recognized as one of the highest priorities. In the meantime, how to develop alternatives to highly toxic cadmium (Cd)-containing materials has become a primary issue in environmentally friendly research. Thus, CIGSe can be used as a less toxic alternative to Cd-containing semiconductors [8]. For large-area fabrication of CIGSe thin-film photovoltaic (PV) modules, the use of sputtering techniques is advantageous [5,8,9]. Due to the preparation of the absorber layer, it is the main core of the CIGSe thin-film solar cell. In this study, copper indium gallium (CIG) precursors will be deposited via a radio frequency (RF) sputtering process. It is then selenized to complete the CIGSe absorber layer of the proposed structure. In the analysis of the overall film structure, this study focuses on the impact of the proportional combination of different metal-CIG-precursor layers on CIGSe films, hoping to realize CIGSe solar cells with absorber layers and different buffer film materials to optimize all sputtering conditions.

However, when the substrate temperature is lower than 500 °C, the diffusion of alkali elements (Na) from the substrate will be limited, thereby reducing the overall efficiency of CIGSe solar cells [10]. In order to solve this limitation, a new approach for the thermal management of solar cells was reported [10,11,12,13], thus improving the crystallinity and grain size of the CIGSe absorption layer deposited for the low-temperature process. Due to their tunable bandgap (from 1.0 eV to 1.7 eV), CIGSe-based chemistries can be used to create high-efficiency thin-film solar cells and are promising absorber materials [14].

In order to elucidate the constraints required to achieve higher efficiency levels, it is also necessary to pay attention to the microscopic loss mechanisms in optoelectronic components. In terms of physics and/or chemistry, however, there are still numerous poorly understood areas that require further development, such as the basic material science and interface defects of CIGSe-based films and devices [15]. Therefore, the two-segment process is designed with two primary objectives: to improve the internal and external quantum efficiencies (IQE/EQE) within the short-wavelength range and to augment the photocurrent and power conversion efficiency (*η*) of CIGSe solar cells on flexible substrates. Beyond these objectives, our investigation extends to the exploration of metal alloy compositions as an alternative to a CdS buffer for environmental sustainability [5,8,9,10,11,12]. Considering environmental awareness and sustainability, the developed eco-friendly PV modules exhibit significant potential for commercial mass production.

## 2. Experiments and Measurements

This study is based on high-PV efficiency solar cells and hopes to conduct research based on open-circuit voltage loss analysis and interface bandgap structure. The main areas include the following: the correlation between the copper (Cu) deficiency phase and the influence of alkali metals, the application of lightweight flexible components, single crystal epitaxial CIGSe films, and devices, etc. A gradient in Ga distribution was also found, and CIGSe was also investigated as an absorber for the top cell in a tandem configuration. As described in our previous work [5,9], the CIGSe cell process mostly used the co-evaporation method to make the absorption layer, the selenization process and the sputtering method to plate the buffer layer and barrier layer, and, finally, the sputtering method to prepare Ni-Al surface electrode.

**Figure 1:**
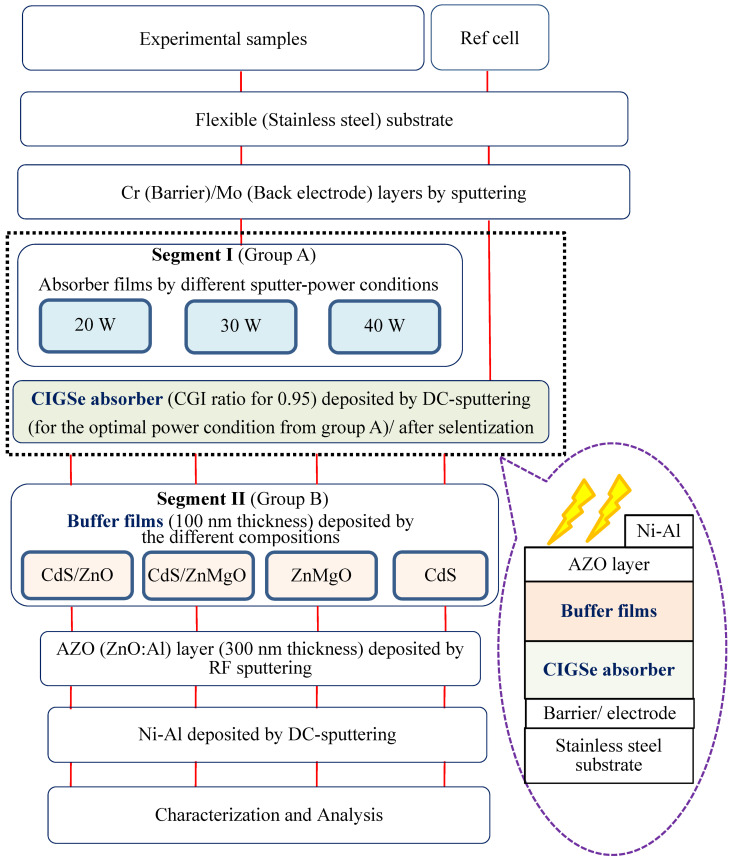
The overall flow chart, and its corresponding schematic device with lighting radiation diagram (inserted) of CIGSe solar cells by two-segment process. In the initial segment (Segment I), carried out under the Cr barrier/Mo back electrode/stainless-steel (flexible) substrate, the absorption layer is fashioned using varied DC sputtering powers. Transitioning to the subsequent segment (Segment II), distinct buffer layers are created, employing diverse micro-chemistry compositions such as CdS/ZnO, CdS/ZnMgO, ZnMgO, and CdS for Group B samples. Finally, for comparative analysis, the AZO (ZnO:Al for 300 nm thickness)/front electrode (Ni-Al) is sputtered to conclude the process.

Figure 1 shows the comprehensive process flowchart and its corresponding schematic diagram (as inserted), detailing the fabrication of CIGSe-based solar cells on flexible substrates through a two-segment process. To identify the optimal sputtering power conditions, firstly, in the segment I process, micron thin-film absorbers were created at different RF sputtering powers/thickness using Cr barrier/Mo back electrode/stainless steel (flexible) substrate at 550 °C. These samples from Group A were 20 W/2.234 μm, 30 W/2.678 μm, and 40 W/2.817 μm for 300 min. Subsequently, in the segment II process aimed at achieving an optimal bandgap, buffers with a thickness of 100 nm were prepared with different micro-chemical compositions (e.g., CdS/ZnO, CdS/ZnMgO, ZnMgO, and CdS for Group B samples). The chemical bath deposition (CBD) was used to prepare a ZnS film on the CIGS absorption layer and then sputtering was used to prepare a ZnMgO film on ZnS as a buffer layer. Thereafter, the completed AZO (ZnO:Al, thickness 300 nm)/front electrode (Ni-Al) was DC sputtered. Finally, all developed schematic structures (at the lower right of Figure 1) were set up for characterization and analysis.

During this selenization process, selenium vapor is used to replace sulfur in the CIGS film [9], which improves the film’s crystallization and grain size. Among them, the CIG film will produce a large number of second phases, such as Cu_2_Se, In_2_Se, In_2_Se_3_, Ga_2_Se, Ga_2_Se_3,_ and other compounds. After selenization is completed, Cu_2_Se is a liquid low-resistance substance that will be formed on the surface of the film. This material creates leakage current paths in the CIGS film, so the growth of secondary phases must be suppressed. The absorber layer was treated with potassium cyanide (KCN) before measurement. At the same time, the selenization temperature is also a key factor that determines the performance of solar cells. As the selenization temperature increases in this study, the film becomes smoother and denser. Therefore, this study uses two different selenization temperatures (550 and 580 °C) and two different selenium parameters to explore the deposition and crystal growth conditions of the absorber layer.

In this study, a ZnMgO thin film was sputtered on the CBD-coated CdS layer as a buffer layer structure. ZnMgO thin films are prepared from ZnMgO ternary alloy targets through RF sputtering. Since it is difficult to analyze the buffer layer structure formed by two different substances, a ZnMgO film was sputtered on the pretreated substrate. In order to explore the structure of the ZnMgO film after sputtering, the Mg concentrations of the target components were 0.15 and 0.25, respectively, under the conditions of film working pressure (2.3 mTorr), substrate rotation speed (20 rpm), and 99.9% Ar working gas.

Surface characterization of the CIGSe film employed an atomic force microscope (AFM, Bruker INNOVA SPM, Santa Barbara, CA, USA). A scanning area of 2.5 × 2.5 mm^2^ (512 × 512 pixels) underwent analysis on a vibration-free platform. Root mean square (RMS) surface roughness values (*R*q) were determined using the software included with the AFM instrument. Furthermore, the cells’ optical and electrical properties underwent evaluation using various commercially available systems, including scanning electron microscopy (SEM) and energy-dispersive X-ray spectroscopy (EDS, Model JSM-6500F, Japan Electron Optics Laboratory (JEOL) Co., Ltd, Tokyo, Japan) instrument. A power conversion efficiency measuring system (Oriel-91192/AM 1.5 GMM) and comprehensive IQE and EQE measurements (Model QE-R, Enlitech Co., Kaohsiung City, Taiwan) were also utilized. The determined CGI optimal ratio for the composite ratio film stood at 0.95, consistent with findings reported in existing literature [9].

## 3. Results and Discussion

In this study’s fabrication of CIGSe solar cells, at first, the polycrystalline CuInGa (CIG) precursors were co-sputtered by Cu, In, and Ga elements on a Cr/ Mo/ flexible (stainless steel) substrate. Among them, different DC sputtering powers are as follows: sample A1 is 20 W, sample A2 is 30 W, and sample A3 is 40 W. In order to explore the surface morphologies of CIG precursors, the sputtering durations were changed by 100, 300, and 600 min. Table 1 illustrates the sputtering parameters of time duration at 80 sccm argon (Ar) flow, 2.3 mtorr working pressure, and 6 rpm rotating speed, alongside the corresponding SEM images for CGI films. Among them, there are defects such as a large amount of agglomeration (duration 100 min) and defects causing a two-layer appearance (duration 600 min) due to agglomeration. The CIG precursor with a sputtering time of 100 min has a stable surface morphology. Thus, it is the surface roughness effect of the CGI films at a given sputtering time (duration 300 min), the same argument found in [12], which is related to its growth rate leading to the formation of uniform micro-particles.

In order to study the characteristics of the three samples with different absorption layers, Table 2 summarizes the elemental composition of the material surface and the corresponding energy-dispersive X-ray spectroscopy (EDS) spectral images. These combined data will be useful in studying the selenization properties of CIGSe absorbers.

This comprehensive data aids in investigating the selenization characteristics of the CIGSe absorbers. Before the selenization process (upper half of Table 2), samples A1 (20 W), A2 (30 W), and A3 (40 W) exhibited CGI ratios ([Cu]/([Ga] + [In]) of 0.812, 1.055, and 0.864, respectively. After selenization (lower half of Table 2), these ratios shifted to 0.649, 0.950, and 0.634, respectively. For the selenization phenomenon, the CGI ratio of sample A2 changes from 1.055 (before) to 0.950 (after), showing better crystal thickness and less secondary phase signal [5,9,10,11]. At the same time, the GGI ratio ([Ga]/([Ga] + [In])) of all samples in the table is in the range of 0.293–0.365, confirming that CIGSe is a high-efficiency component. The content of Ga atoms (between 0.1 and 0.4) confirms that CIGSe is a high-efficiency component [9,10]. This delineates a notable potential for enhancing the crystallinity of CIGSe films deposited at lower temperatures, exemplifying a promising procedure.

**Figure 2:**
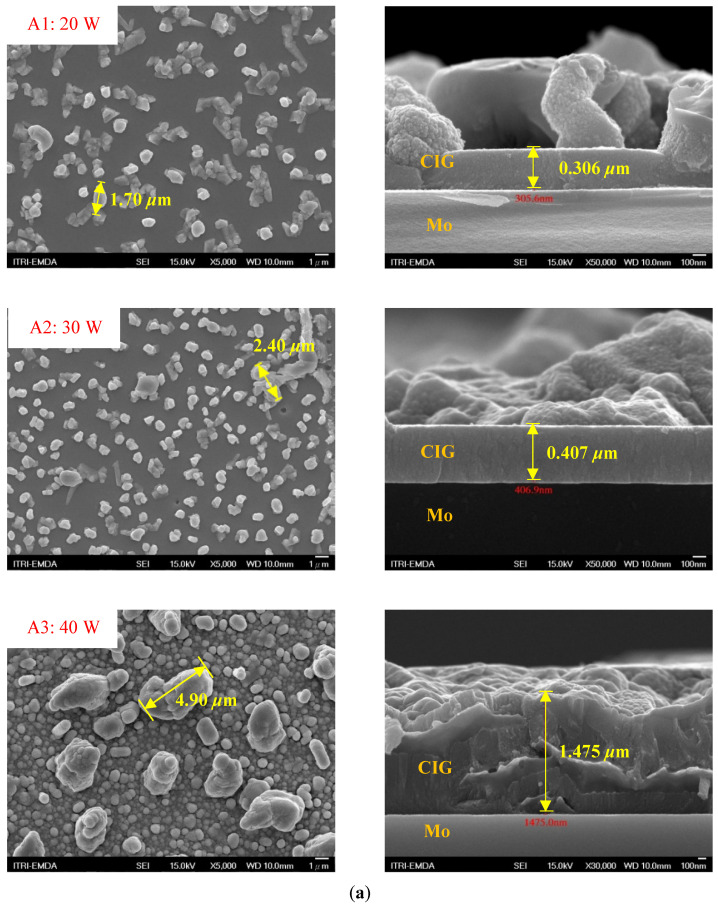

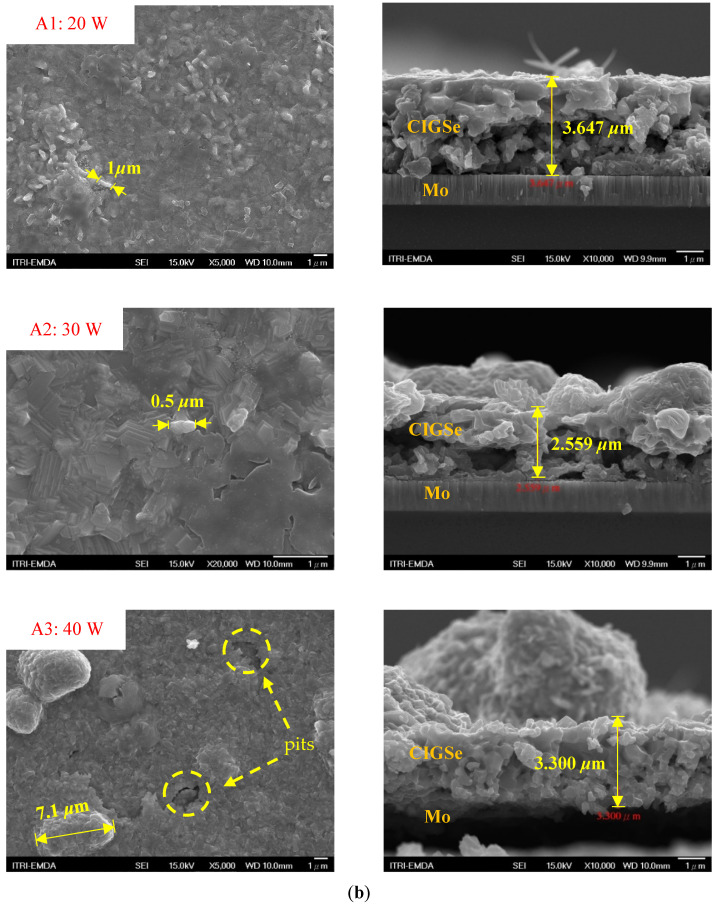
The SEM images depict the CIGSe absorber microfilms post-segment I processing. The illustrations include both top-view (**left**) and cross-sectional perspectives (**right**), each generated at distinct power settings—A1: 20 W, A2: 30 W, A3: 40 W. (**a**) Before selenization, images showcase particulate sizes ranging from 1.7 to 4.9 μm, correlating with thickness variations spanning from 305.6 to 1475 nm. (**b**) After selenization, the particulate sizes are observed at 1 μm (A1), 0.5 μm (A2), and 7.1 μm (A3), aligning with thickness measurements of 3.647 μm (A1), 2.559 μm (A2), and 3.3 μm (A3), respectively.

Figure 2 presents SEM images providing top (left) and cross-sectional (right) perspective views of the CIGSe absorber after the segment I process at different sputtering powers (A1: 20 W, A2: 30 W, A3: 40 W). These images capture the stages (a) before selenization and (b) after selenization. It is worth noting that visual inspection revealed a significant increase in grain size when operating at higher sputtering power, which was evident in both the pre- and post-selenization stages. Despite the presence of microfilm, the surface morphology of each CIGSe absorber is predominantly characterized by the underlying particulate structure [14]. Before selenization, the images of the CIG film in Figure 2a display numerous lumpy structures, with the particulate size progressively increasing with the incremental rise in sputtering power (20–40 W). The subsequent selenization of the CIG film, particularly at higher sputtering power (40 W), as depicted in Figure 2b, results in the formation of uneven pits across the film’s surface.

Due to the KCN etching in the selenization process, the surface of the CIGSe film will become rough, which will have an impact on subsequent processes. In order to analyze the changes in deposition after electroplating the absorber layer, it can be seen from the top view of Figure 2b that there are obviously many block structures on the surface of the film. The original particles of the CIG film have been transformed into a smoother surface after being selenized. After the CIG film is selenized under low sputtering power (20 W), the surface of the film is relatively smooth and dense. Relatively, under high sputtering power (40 W) observed from the cross-section, the surface of the film is uneven, forming large particles and cavities. It can be found from the figure that, especially at high power, the crystal growth of the film after selenization is not good.

The absorber thickness of sample A1 (0.306 μm in right—Figure 2a) is notably thinner than that of sample A3 (1.475 μm in right—Figure 2a). Moreover, quantification from the EDS spectra of CIGSe films distinctly identifies sputtering powers of 20, 30, and 40 W for samples A1, A2, and A3, respectively, as outlined in Table 2. Upon the selenization of the CIG film, a slower reaction and growth rate occur, resulting in the formation of uniform nanoparticles [14,15,16]. Furthermore, the incorporation of Cu-rich content, notably observed in sample A2 with a 0.95 CGI ratio, within the CIGSe absorber microfilm distinctly promotes grain growth. This diminishes the presence of fine-grain film, significantly enhancing absorber crystallinity and concurrently reducing trap state density [17].

Table 3 presents 2-D and 3-D AFM images of CIGSe thin-film absorbers manufactured through the segment I process. All AFM results were obtained by performing multiple scans at different locations on each sample. The average values of *R*a and *R*max in samples A1 (20 W), A2 (30 W), and A3 (40 W) were recorded separately as (a) 17.0/366, 71.8/480, and 80.0/520 nm before selenization, and (b) 40.2/336.0, 10.5/320.0, and 379.5/682.6 nm after selenization. Here, *R*max represents the maximum surface height, while Ra denotes the average center-line roughness. In the A2 image after selenization (Table 3b), from taking multiple scans over different locations on each sample, an apparently dense and flat surface structure composed of tapered pillars was observed at the average values of *R*a (10.5 nm) and *R*max (320.0 nm), comprising cone-shaped columns randomly distributed across the film’s surface. Consequently, the film exhibits a rough and porous surface with an RMS surface roughness (*R*q) of 23.0 nm. These observations are consistent with the previous literature [18,19,20], emphasizing that the porous film structure achieved using 30 W sputtering power produces the smallest grain size (~0.5 μm), as imaged in Figure 2.

As previously highlighted, the primary objective of this research is to enhance the overall efficiencies of *η*, IQE, and EQE values of developed CIGSe cells in the ultraviolet (UV)–visible wavelength region. Upon choosing the optimal 30 W CIGSe absorber sputtered by the segment I process, we focus on aligning the CIGSe absorber/buffer bandgap diagrams to maximize light absorption, thereby minimizing light loss [6]. Moving into the subsequent segment II process, the buffer layers (100 nm thickness) encompass diverse micro-chemistry compositions (CdS/ZnO, CdS/ZnMgO, ZnMgO, and CdS for Group B samples). Notably, the electrical resistivity of the microfilm increases with magnesium (Mg) content [21]. The fabrication of Zn_1−*x*_Mg*_x_*O buffer films through RF sputtering utilizes ternary alloy targets, allowing precise adjustment of Mg content to fulfill the requisite for optical bandgap application. Consequently, the Tauc function serves as the tool for determining the optical bandgap (*E*_g_ in eV) applicable to disordered or amorphous semiconductors [22]. This measurement correlates with the energy and wavelength of the photoelectron generated when a material is exposed to incident light, following the principles outlined in the photoelectric effect equation [23], expressed as

**Figure 3:**
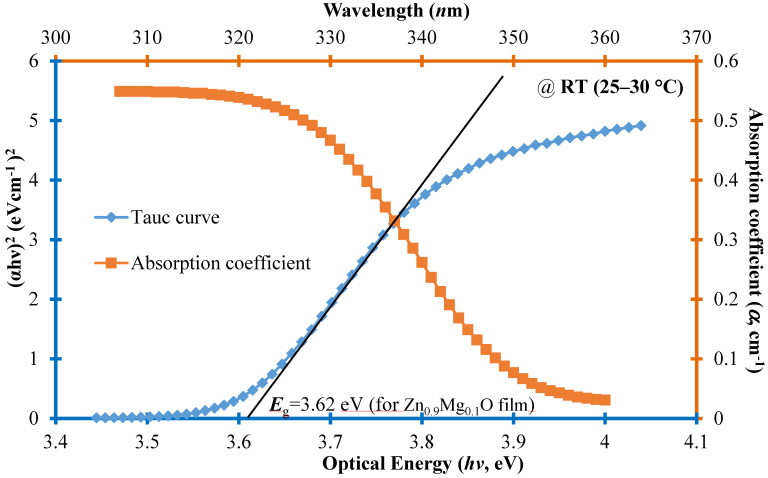
The optical band diagram (*hν* in eV) of the Zn_1−*x*_Mg*_x_*O thin film is presented alongside a comparative Tauc curve [(α*h*ν)^2^ in (eV cm^−1^)^2^, bottom-left axis] and absorption coefficient (*α* in cm^−1^, correlated with wavelength on the top-right axis) of CIGSe solar cells. Notably, for the Zn_1−*x*_Mg*_x_*O film with Mg content (*x* = 0.1), the energy bandgap (*E*_g_) is determined to be 3.62 eV.

(1) αhν12=Ahν−Eg,

For Zn_1−*x*_Mg*_x_*O thin film [21],

(2) $$E_{g}=3.37+2.51\left(x\right)$$

where *α* is the absorption coefficient; *A* is a constant reflecting the extent of band tailing; and *hν* is the incident photon energy. The energy bandgap of the Zn_1−*x*_Mg*_x_*O film, associated with varying Mg concentrations, can be calculated using Equation (2). Figure 3 illustrates a comparative analysis of the optical energy diagram (*hν* in eV) of the Zn_1−*x*_Mg*_x_*O thin film using the Tauc function (Equation (1)) and absorption coefficient (*α* in cm^−1^), incorporating Mg content (Equation (2)) at room temperature (RT, 25–30 °C) for optimal utilization in optical bandgap applications.

Moreover, the corresponding value of *E*_g_ (measuring 3.62 eV for *x* = 0.1 in the Zn_1−*x*_Mg*_x_*O film) is determined by extrapolating the linear portion of the curve to the *hν* axis, reaching the point where (*αhν*)^1/2^ = 0, as visually depicted in Figure 3. This tunable bandgap range (3.62–5.63 eV) shows the linear dependence of the bandgap on the Mg composition (*x*). This finding is consistent with that reported by Singh et al. [21].

**Figure 4:**
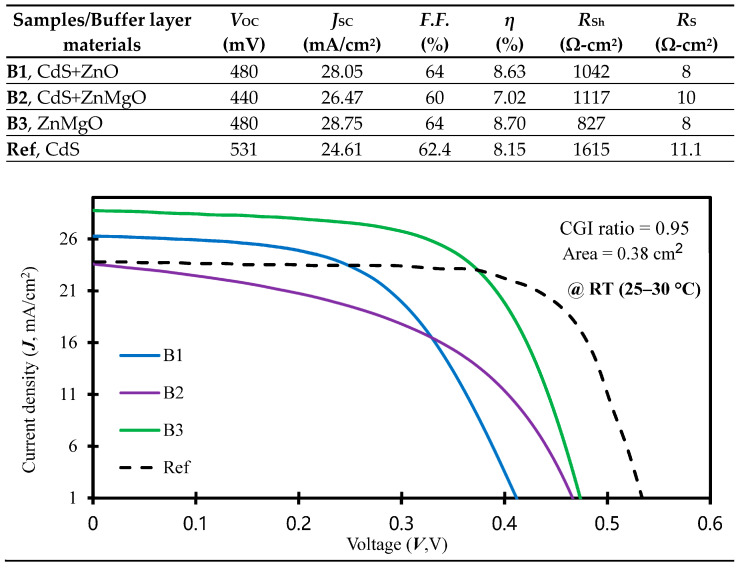
The current density–output voltage (*J*-*V*) curves were generated utilizing a range of buffer layer micro-chemistry materials (CdS/ZnO, CdS/ZnMgO, ZnMgO, and CdS) applied to samples B1, B2, B3, and the Ref cell in RT environment. The opto-electrical performance metrics of the CIGSe solar cells are juxtaposed above their respective curves for analysis.

The current density–voltage (*J*-*V*) characteristics of the CIGSe cells on flexible substrates, optimized with a CGI ratio of 0.95 as per the literature [9], were experimentally examined under AM1.5G illumination conditions with an irradiance of 1000 W/cm^2^ in an RT environment. Figure 4 plots the *J*-*V* curve under illumination, presenting the electrical properties of the CIGSe layer solar cells at the top. The most outstanding performance was observed in sample B3, featuring a ZnMgO buffer material, yielding the highest efficiency for the CGISe layer solar cell. This B3 cell configuration exhibited a short-circuit current (*J*_SC_) of 28.75 mA/cm^2^, an open-circuit voltage (*V*_OC_) of 480 mV, a series resistance (*R*_S_) of 8 Ω-cm^2^, and a remarkable *η* value of 8.70%. The reason is with taking advantage of the multiple-quantum-well (MQW) structure of ZnO/ZnMgO for optimized device performance [24], which also signifies a substantial absolute gain of 1.68% when compared to the B2 cell. Further investigation revealed that the synthesis technique employed for the lower Mg content device, utilizing a Zn_0.9_Mg_0.1_O film, addressed a phenomenon observed with the approximate 3.62 eV bandgap in the CIGSe buffer layer within the optical band spectra (refer to Figure 3). This enhancement in optoelectronic quality notably improves photon penetration into the buffer layer [25].

The two-segment process serves two purposes: enhancing both the IQE and EQE within the short-wavelength range and simultaneously bolstering the photocurrent and *η* value of CIGSe solar cells. In Figure 4, we employed the short-circuit current density (*J*_SC_), incident light power (*P*_L_), optical bandgap (*E*_g_), and reflectance (*R*(*λ*)) to compute the local IQE concerning wavelength (IQE(*λ*)). This calculation utilized the following equation [26]:

(3) IQEλ=JSCqPLEg11−R(λ)=EQEλ1−R(λ)

**Figure 5:**
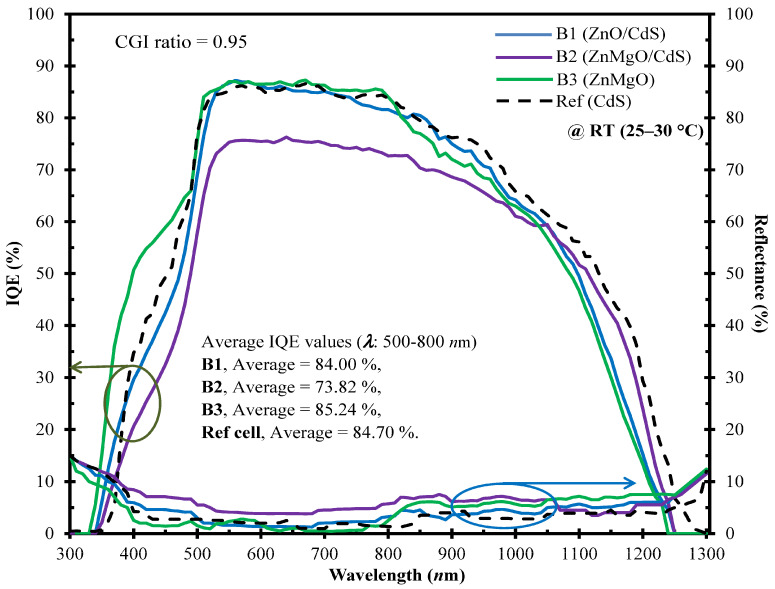
A spectral optoelectronic analysis comparing the internal quantum efficiency (IQE in %, bottom-left axis for the green/arrowed lines) and reflectance (*R*(*λ*) in %, bottom-right axis for the blue/arrowed lines) across wavelengths ranging from 500 to 800 nm is conducted for CIGSe solar cells at ambient RT. These cells, designed with an optimal CGI ratio nearing 0.95, were prepared using distinct buffer layer micro-chemistry materials (CdS/ZnO, CdS/ZnMgO, ZnMgO, and CdS) denoted as samples B1, B2, B3, and the Ref cell.

Figure 5 presents the IQE values (calculated using Equation (3)) alongside the corresponding reflectance (*R*(*λ*) measured in an RT environment across the 300–1300 nm wavelength radiation) for various micro-chemistry compositions (B1: CdS/ZnO, B2: CdS/ZnMgO, B3: ZnMgO, and Ref: CdS). The IQE curve of sample B3 (ZnMgO buffer cell, averaging 85.24%) surpasses those of other cells within the visible range (500–800 nm wavelength). Additionally, the measured *R*(*λ*) and IQE values are concurrently fitted to accommodate the dependence of electrical performance on the optical properties (as depicted in the top chart of Figure 4) of the cell. For sample B3, the *J*_SC_ value correlates with the buffer film bandgap, with a higher *J*_SC_ value aligning with the Mg content composition, resulting in elevated *η* values. This increase in the IQE leads to decreased reflectance in the longer wavelength range. The decline in the IQE observed at shorter wavelengths (below 500 nm) can be ascribed to surface recombination, aligning with the conclusions drawn in the study conducted by Cheng et al. [27].

**Figure 6:**
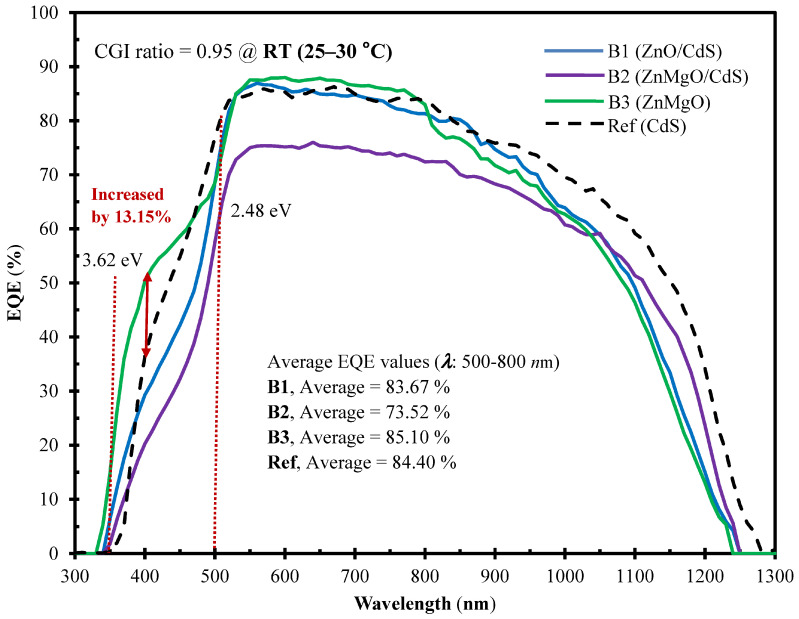
At consistent ambient RT, the external quantum efficiency (EQE in %) of CIGSe solar cells prepared with different buffer layer micro-chemistry materials (CdS/ZnO, CdS/ZnMgO, ZnMgO, CdS) for samples of B1, B2, B3, and Ref cell. Within the short-wavelength range (350–500 nm, corresponding to band gaps of 3.62–2.48 eV for the brown dotted/arrowed lines), the average EQE value of sample B3 surpassed that of the Ref cell by 13.15%.

Figure 6 illustrates spectral EQE curves across a broad wavelength span (300–1300 nm) for the three developed samples alongside the Ref cell in an RT environment. The EQE values of the developed samples consistently fall below those of the Ref cell across all wavelengths. Within the short-wavelength range (350–500 nm, corresponding to bandgaps of 3.62–2.48 eV), the average EQE value of sample B3 surpassed that of the Ref cell by 13.15%. This particular enhancement underscores the effectiveness of the bandgap (*E*_g_ = 3.62 eV from Figure 3) achieved through the optimal Zn_0.9_Mg_0.1_O content buffer. Additionally, the incorporation of Mo as a back surface layer serving as a hole transport-electron reflected layer contributes to this observed improvement [3].

In the extended wavelength range spanning from 500 to 800 nm, the average EQE values stand at 83.67%, 73.52%, 85.10%, and 84.40% for samples B1, B2, B3, and the Ref cell, respectively. This range corresponds to a Ga-grading bandgap at the back side, where Mo serves the crucial function of providing effective self-passivation at the absorber/back contact interface [28]. This signifies a distinct enhancement in self-passivation quality, enabling quasi-ohmic electrical contact that effectively counters the Mo diffusion effect on CIGSe/Mo interfaces. Sample B3 exhibits the most favorable average across the curve (85.10%), correlating with the highest fill factor (*F*.*F*.) and the peak conversion efficiency (*η* = 8.70%), as shown in Figure 4. Within the proposed two-segment process, we have optimized overall efficiency performance by fine-tuning cell parameters concerning RF sputtering powers for the CIGSe absorber layer and the Mg concentration within the Zn_1−*x*_Mg*_x_*O buffer film. This Cd alternative buffer layer not only improves light absorption [29] but also satisfies the environmental requirement for Cd-usage reduction.

**Figure 7:**
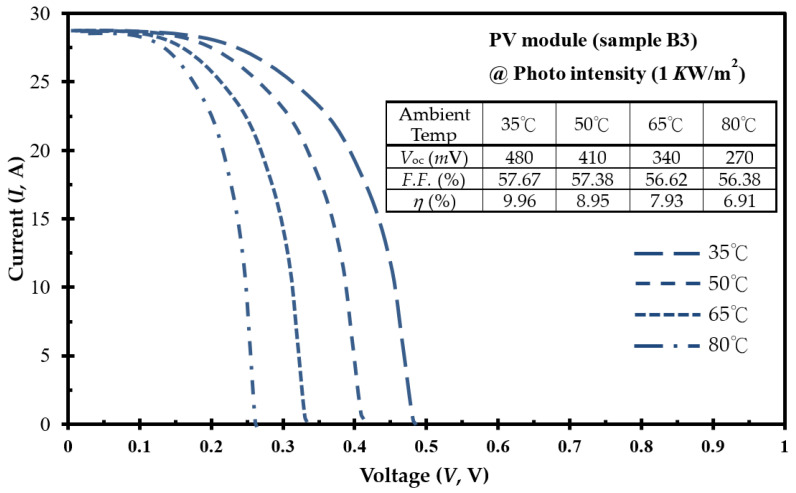
Under a constant irradiance level (KW/m^2^), the current–voltage (I–V) curves were captured for a PV module consisting of nine optimal CIGSe solar cells (sample B3) at varied ambient temperatures (35–80 °C). Additionally, the opto-electrical performance of the PV system is depicted in the top inset.

Constructed from nine optimal CIGSe solar cells (sample B3) for the dimension of 30 × 30-cm^2^ area, the PV array module was connected to a variable load (rheostat of 220 Ω, and 10 A) through multi-meters for current and voltage measurement. Figure 7 illustrates the current (A)–voltage (V) curves of a PV module under a photo-intensity of 1 KW/m^2^ and reveals the impact of varying ambient temperatures. *V*_OC_ decreases from 480 to 270 mV as the ambient temperature rises from 35 to 80 °C. The corresponding opto-electrical performance parameters (*V*_OC_, fill factor (*F*.*F*.), and *η* values) for the developed PV module at these diverse ambient temperatures are shown in the upper portion of Figure 7. As the temperature increases, the optical bandgap of the semiconductor contracts, leading to a reduction in the *V*_OC_ value. This conforms to the temperature-dependent behavior of the *p*–*n* junction voltage, as elucidated by the Tauc function in Equation (1), which explains the negative temperature effect on the *V*_OC_ value. This reduction consequently contributes to a decline in the *η* value, dropping from 9.96% to 6.91%, aligning with similar observations previously reported in [9].

**Figure 8:**
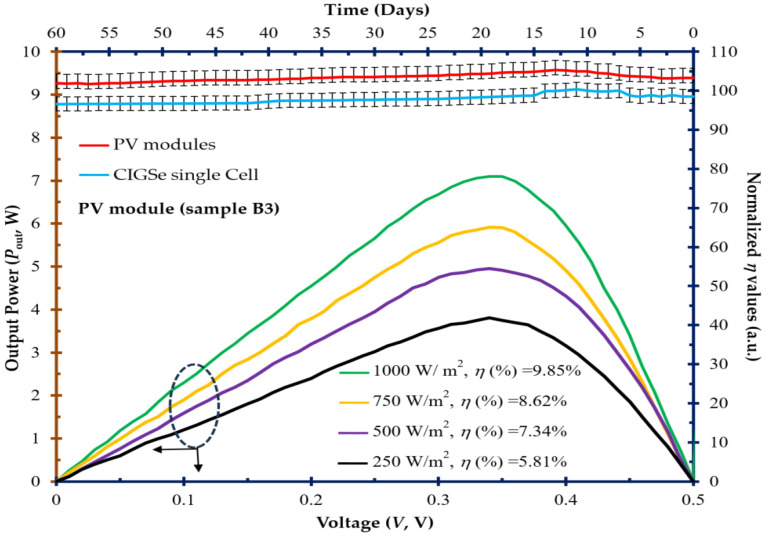
At consistent ambient temperature of 25 °C, output power–voltage (*P*_out_-V) curves were produced for the optimal CIGSe solar cells assembled within a PV module (sample B3). These curves were observed across a spectrum of photo-intensities (250–1000 W/m^2^) [bottom-left axis with the dotted circle and arrows]. Moreover, stability curves portraying normalized efficiency values (*η*) (a.u.) are presented, corresponding to time (days) on the top-right axis, for both the PV module and an individual CIGSe solar cell under an irradiance level of 1000 W/m^2^.

At a steady ambient temperature of 25 °C, the optimal CIGSe solar cells housed within a PV module (sample B3) reveal a discernible, linearly increasing relationship between output power and voltage (*P*_out_-V), as depicted in Figure 8. This trend becomes apparent with a gradual elevation in photon intensity ranging from 250 to 1000 W/m^2^. Conversely, this illustrates the positive impact of photo-intensity on the *V*_OC_ value, resulting in an enhancement of its *η* value (from 5.81% to 9.85%). This observation aligns with the findings reported in [30].

Concurrently, Figure 8 shows the stability curve detailing the normalized *η* values evaluated over a duration of 60 days. These values were standardized against the initial performances of the as-grown cell/module without any adjustments for irradiance levels [31]. Consequently, the cyclic patterns corresponding to day and night cycles are discernible within the dataset. The error bars associated with each data point represent the range between the maximum and minimum values observed for both the PV module and CIGSe single cell at each specific data point. Remarkably, these variations oscillate within a margin of less than ±5% as seen on the top-right axis of Figure 8. This consistency underscores the acceptable stability of *η* values exhibited by the developed PV module and the individual CIGSe cell—an argument that aligns with findings in the existing literature [32].

## 4. Conclusions

In summary, an intricate two-segment process was meticulously developed and detailed for the CIGSe solar cells on flexible substrates. The initial phase, segment I, involved fine-tuning the DC sputtering powers within the CIGSe absorber process, ranging from 20 W to 40 W. Opting for the optimal 30 W CIGSe absorber (featuring a 0.95 CGI ratio) yielded significant benefits—specifically, the Cu-rich film’s remarkable ability to stimulate grain growth and reduced trap state density. Moving to segment II, the CIGSe buffer process entailed exploring various micro-chemistry compositions (CdS, ZnO/CdS, ZnMgO/CdS, and ZnMgO) through optical bandgap semiconductor theories. Following this segment II process, an eco-conscious shift away from toxic CdS was achieved. The Zn_0.9_Mg_0.1_O alloy buffer emerged as a standout, attaining the highest conversion efficiency (*η* = 8.70%).

In the context of PV modules featuring nine optimal CIGSe solar cells (CGI = 0.95) intended for commercial mass production, the overall performance, as measured by the IQE and EQE, exhibits significant variations across different levels of photo-intensity. These variations in incident solar radiation on the PV module influence multiple parameters, including *V*_OC_, *F*.*F*., *η*, and overall output power. Heightened light intensities distinctly correlate with the augmented power generation capabilities of the solar cell. Our objective is to not only enhance the performance of the developed devices but also to reduce process temperatures and production costs, and lessen the use of toxic Cd-containing materials. In adherence to sustainable development objectives, the integration of eco-friendly PV modules emerges as a promising avenue for seamless integration into commercial mass production.

## Figures and Tables

**Table 1:** The sputtering process with time duration of 80 sccm Ar flow and 6 rpm rotating speed is presented alongside the corresponding SEM images for CIG films. These films were prepared by varying sputtering powers: 20 W for sample A1, 30 W for sample A2, and 40 W for sample A3.

CIG Sputtering Time (min) (Ar Flow: 80 sccm, Rotating Speed: 6 rpm)							
	Powers	20 W (A1)		30 W (A2)		40 W (A3)	
Thickness							
100 min		0.129 μm	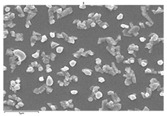	0.211 μm	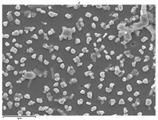	0.437 μm	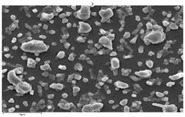
300 min		0.306 μm	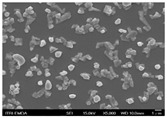	0.407 μm	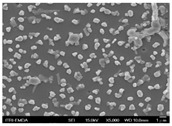	1.475 μm	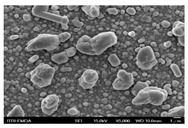
600 min		1.434 μm	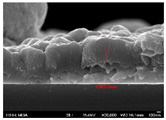	2.178 μm	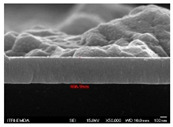	2.902 μm	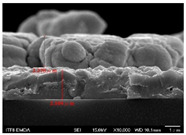

**Table 2:** The surface compositions, both before and after the selenization process, are presented alongside the corresponding images of EDS spectrum for CIGSe absorber films. These films were prepared by varying sputtering powers: 20 W for sample A1, 30 W for sample A2, and 40 W for sample A3.

Atomic Ratio (%)		Cu K	In L	Ga K	Se L	CGI Ratio	GGI Ratio
Before selenization	A1 (20 W)	44.80	36.37	18.53	0	0.812	0.341
	A2 (30 W)	51.33	31.77	16.90	0	1.055	0.347
	A3 (40 W)	46.36	37.94	15.70	0	0.864	0.293
After selenization	A1 (20 W)	20.31	20.00	11.29	48.38	0.649	0.361
	A2 (30 W)	24.90	16.13	10.03	48.94	0.950	0.365
	A3 (40 W)	20.27	21.47	11.09	48.45	0.634	0.339
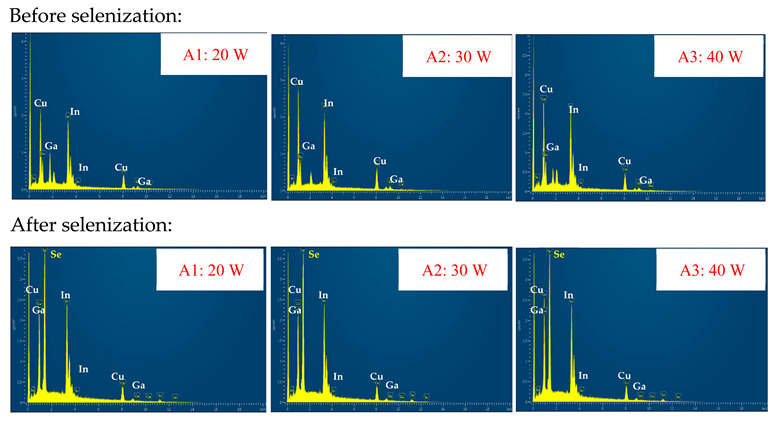							

**Table 3:** The AFM roughness of CIGSe thin-film absorption layer prepared by segment I with RMS (*R*q) values. (**a**) Before selenization, samples A1 (20 W), A2 (30 W), and A3 (40 W) for 25.9, 88.2, and 108.2 nm, respectively. (**b**) After selenization, samples A1 (20 W), A2 (30 W), and A3 (40 W) for 48.4, 23.0 and 389.6 nm, respectively.

Before Selenization	RMS (*R*q)	*R*a	*R*max
A1, Sputter power 20 W	25.9 nm	17.0 nm	366 nm
A2, Sputter power 30 W	88.2 nm	71.8 nm	480 nm
A3, Sputter power 40 W	108.2 nm	80.0 nm	520 nm
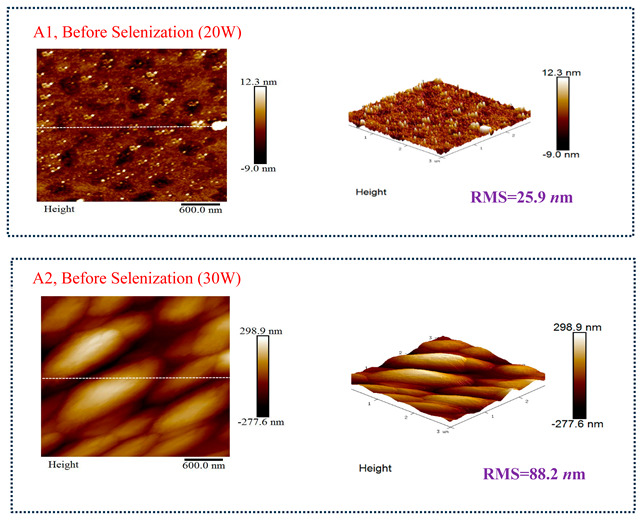 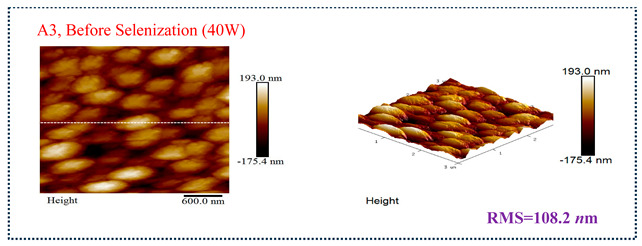			
(**a**)			
**After Selenization**	**RMS (*R*q)**	***R*a**	***R*max**
A1, Sputter power 20 W	48.4 nm	40.2 nm	336.0 nm
A2, Sputter power 30 W	23.0 nm	10.5 nm	320.0 nm
A3, Sputter power 40 W	389.6 nm	379.5 nm	682.6 nm
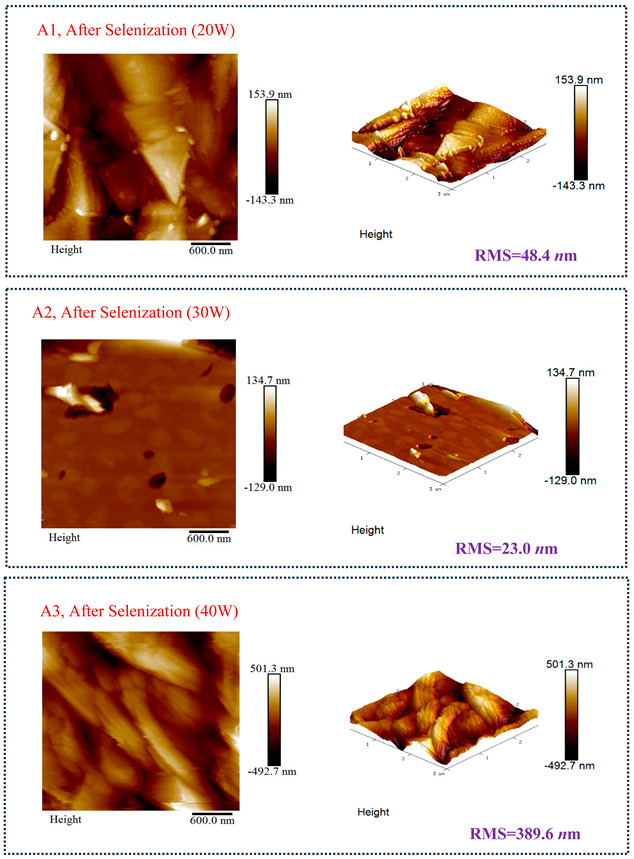			
(**b**)			

## Data Availability

The data presented in this study are available on request from the corresponding author. The data are not publicly available due to privacy and ethical restrictions.
